# Dosimetry of interface region near closed air cavities for Co-60, 6 MV and 15 MV photon beams using Monte Carlo simulations

**DOI:** 10.4103/0971-6203.62197

**Published:** 2010

**Authors:** Chandra P. Joshi, Johnson Darko, P. B. Vidyasagar, L. John Schreiner

**Affiliations:** 1 Cancer Centre of South Eastern Ontario at Kingston General Hospital, 25 King Street West, Kingston, ON, K7L5P9, Canada; 2 Departments of Oncology and Physics, Queen's University, Kingston, ON, Canada; 3 Department of Physics, University of Pune, Pune-411 007, India

**Keywords:** Air cavity, electronic disequilibrium, interface dosimetry, % dose reduction

## Abstract

Underdosing of treatment targets can occur in radiation therapy due to electronic disequilibrium around air-tissue interfaces when tumors are situated near natural air cavities. These effects have been shown to increase with the beam energy and decrease with the field size. Intensity modulated radiation therapy (IMRT) and tomotherapy techniques employ combinations of multiple small radiation beamlets of varying intensities to deliver highly conformal radiation therapy. The use of small beamlets in these techniques may therefore result in underdosing of treatment target in the air-tissue interfaces region surrounding an air cavity. This work was undertaken to investigate dose reductions near the air-water interfaces of 1×1×1 and 3×3×3 cm^3^ air cavities, typically encountered in the treatment of head and neck cancer utilizing radiation therapy techniques such as IMRT and tomotherapy using small fields of Co-60, 6 MV and 15 MV photons. Additional investigations were performed for larger photon field sizes encompassing the entire air-cavity, such as encountered in conventional three dimensional conformal radiation therapy (3DCRT) techniques. The EGSnrc/DOSXYZnrc Monte Carlo code was used to calculate the dose reductions (in water) in air-water interface region for single, parallel opposed and four field irradiations with 2×2 cm^2^ (beamlet), 10×2 cm^2^ (fan beam), 5×5 and 7×7 cm^2^ field sizes. The magnitude of dose reduction in water near air-water interface increases with photon energy; decreases with distance from the interface as well as decreases as the number of beams are increased. No dose reductions were observed for large field sizes encompassing the air cavities. The results demonstrate that Co-60 beams may provide significantly smaller interface dose reductions than 6 MV and 15 MV irradiations for small field irradiations such as used in IMRT and tomotherapy.

## Introduction

Cancer can often occur in the close vicinity of natural or artificial air filled spaces in the human body. Such air-filled spaces include closed cavities such as ethmoid, sphenoid and maxillary sinuses in the head and neck (H and N) region, and longitudinal air channel in the trachea. In radiation treatment of H and N cancers, the presence of accessories such as a mouth bite with an air tube for breathing purposes can also often create large air cavities.

It has long been known that the presence of air-filled spaces create conditions of electronic disequilibrium near air-tissue interfaces.[1–6] This phenomenon results in radiation dose build-down and build-up near proximal and distal air-tissue interface regions, respectively. Thus, an increased risk of recurrence of cancer may exist near air-tissue interfaces. The dose reduction (i.e. underdosing) has been shown to increase with the beam energy and decrease with the size of the radiation field.[2–6] Intensity modulated radiation therapy (IMRT) and tomotherapy techniques employ a combination of multiple small radiation beams and beamlets of varying intensities to deliver highly conformal radiation therapy.[7,8] Significant potential exists for underdosing the clinical target volume (CTV) in air-tissue interface regions when these techniques are used for treatment where the CTV is either wrapped around or situated in close vicinity to an air cavity.

Waldron *et al,*[9,10] reported significant local recurrence rate of malignant disease in two separate retrospective trials involving 29 ethmoid sinus and 110 maxillary antrum cancer cases treated with curative intent. In these studies they expressed concern about the risk of local control of disease due to potential underdosing of the target because of the physical uncertainties of the dose distribution achieved in irradiating large air cavities.[9,10]

The problem of electronic disequilibrium and underdosing near air-tissue interfaces is well known and has been reported since the introduction of mega-voltage photon energies in radiation therapy. In 1958, Epp *et al*, drew attention to the possible underdosing caused by ionization build-up in upper respiratory air passages with Cobalt-60 (Co-60) external beam therapy.[1] Investigations by Mohan *et al* and Mackie *et al,* showed that contemporary dose computation algorithms could incorrectly calculate the dose in the regions of electronic disequilibrium.[11,12] More recently, Li *et al,* reported that dose perturbations near air-tissue interface are strongly dependent on x-ray energy, field size, depth, and size of the air cavity.[2] They showed that an underdosing of 42% and 21% can occur at 0.05 mm from an air-tissue interface which they created using a 3 cm thick air slab and irradiating it with the single 5×5 cm^2^ field of 15 MV photons. Wadi-Ramahi *et al,* suggested that a uniform longitudinal magnetic field of 0.5 T strength could be used to reduce secondary electron out-scatter caused by the presence of an air gap to improve the dose at the distal surface of air cavities.[3,4]

Majority of the above investigations on the dose reductions near air-tissue interfaces are representative of open ended longitudinal air gaps such as created by the trachea in the head and neck regions and by air filled rectal balloons in prostate cases.[1–5] Results of most of these investigations are based on dose measurements in the electronic disequilibrium region distal to the air gaps. Limited research data are available on interface dosimetry near closed air cavities probably due to the immense challenges associated with the dose measurements in interface region around closed air cavities.

Schreiner *et al,* have proposed a Co-60 based tomotherapy dose delivery and suggested that it can be a clinically and commercially viable alternative to 6 MV linac based IMRT approaches.[13] Further work by Cadman[14] and Joshi *et al,*[15] showed the potential for Co-60 based tomotherapy. Recently, Fox *et al,*[16] have also demonstrated that Co-60 based IMRT can achieve nearly identical plans compared to 6 MV IMRT.

The work presented in this study was also undertaken to explore potential clinical benefits for Co-60 IMRT and tomotherapy in relation to air-tissue interface doses. The rationale for this is derived from the published literature suggesting that interface doses for small beam sizes improve with decreasing photon energy. The use of Co-60 beamlets for tomotherapy with their lower photon energy may provide dose improvement at air-tissue interfaces compared to 6 and 15 MV beams. In this work, the EGSnrc/DOSXYZnrc Monte Carlo code[17,18] was used to investigate the dose reduction in water near air-water interfaces of closed cubic air cavities for irradiation techniques using small radiation beams or beamlets such as encountered in IMRT and tomotherapy. We have considered air-water interface doses as representative of air-tissue interface doses. Conventional three dimensional conformal radiation therapy (3DCRT) techniques using large field sizes defined by poured blocks and MLCs are still widely used worldwide. To address concerns regarding dose reductions near air interfaces in conventional 3DCRT techniques, and for the completeness of this work, interface dose reductions in water near air-water interfaces were also investigated for a larger field size encompassing the air-cavity.

## Materials and Methods

**Figure 1:**
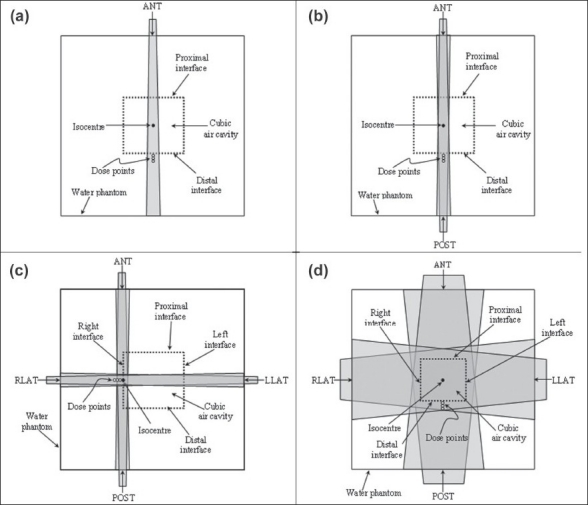
The beam and cavity geometries used for the various Monte Carlo simulations in this study. (a) Single field (SF), (b) Parallel opposed (POP) fields, (c) Four fields (4F)-small fields (2×2 and 10×2 cm^2^) and (d) 4F- large fields (5×5 or 7×7 cm^2^). Note: The centers of air cavities (1×1×1 or 3×3×3 cm^3^) coincide with the centre of the 20×20×20cm^3^ water phantom. All beams are symmetrical and their central axes pass through the isocenter. The “Dose points” indicated to represent the location of the interface dose calculation points (at 0.25, 0.75 and 1.25 mm from the interface (not to scale)).

The 1×1×1 cm^3^ and 3×3×3 cm^3^ air-cavity volumes represent typical medium and large sized cavity volumes in the head and neck region such as presented by ethmoid and maxillary sinuses. In this study, 1×1×1 cm^3^ and 3×3×3 cm^3^ air-cavities in a 20 × 20 × 20 cm^3^ water phantom were modeled [Figure 1] using the EGSnrc/DOSXYZnrc Monte-Carlo (MC) code[17,18] The centers of these air cavities coincide with the center of the phantom for all simulation geometries [Figure 1]. Monte Carlo dose calculations in the interface region of these cubic air cavities were performed using DOSXYZnrc code.[17,18] In the interface region surrounding the cavities, a matrix of voxel size 0.05×0.5×0.5 cm^3^ (volume 0.0125 cm^3^) was chosen for dose scoring at sub-millimeter distances from the air-water interface; the dimension 0.05 cm represents the voxel dimension along a plane perpendicular to the cavity edge. Voxel dimensions of 0.5 cm in the planes parallel to the cavity edges were used to maintain an adequate scoring volume that ensured ≤ 1% statistical uncertainty in dose scoring within reasonable computation times. MC simulations were performed for a single field (SF), parallel opposed field (POP) and four field (4F) irradiations with Co-60, 6 MV and 15 MV photon energies. Dose calculations were performed for beam sizes of 2×2 cm^2^ (beamlet) and 10×2 cm^2^ (fan beam), and 5×5 cm^2^ and 7×7 cm^2^ (i.e. large beams). Dose calculations were also performed for 5×5 cm^2^ and 7×7 cm^2^ beams for 1×1×1 cm^3^ and 3×3×3 cm^3^ air-cavities, respectively (the beam sizes include 2 cm margins around the air cavities). These calculations were undertaken to evaluate dose reduction near interfaces in conventional 3DCRT irradiations.

The global electron cut-off energy (ECUT) and global photon cut-off energy (PCUT) of 0.521 MeV and 0.01 MeV, respectively, were used in all simulations. Published 6 MV and 15 MV[19] and Co-60[20] photon spectra were used as input in the simulations to generate the radiation fields within the DOSXYZ code. Typically, between 1×10^9^ and 20×10^9^ histories were used in the MC simulations to achieve statistical errors ≤1%. All simulations were repeated in a homogeneous water phantom with identical voxel matrices to provide the reference doses for comparison. From the MC calculated dose data, percentage dose reductions (%DR) at sub-millimeter distances (at 0.025 cm, 0.075 cm, 0.125 cm etc.) from the air-water interface were obtained for different beam sizes, cavity sizes, and techniques. The locations of these three dose data points are illustrated in Figure 1. The percentage dose reduction (%DR) at a point represents the percentage reduction in dose at that point in the presence of air cavity compared to the dose at the same point in a homogeneous water phantom (in the absence of the cavity).

## Results

**Figure 2:**
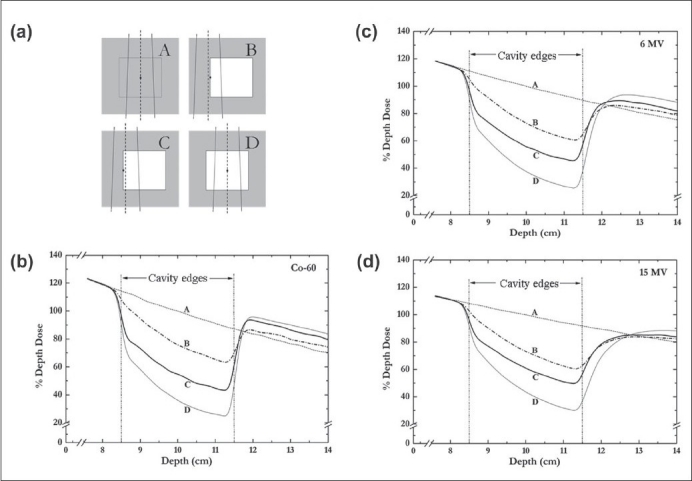
(a) Beam and phantom geometries for calculations of %DD data for 2×2 cm^2^ beamlets of Co-60, 6 MV and 15 MV for conditions A, B, C and D (the external dimensions of the water phantom are truncated and the distances are not to scale). Figures (b), (c) and (d) show the %DD data along the central axis (CAX) of the beamlet for all four conditions. The locations of the CAX, in relation to the air-water interfaces, are represented by the vertical dashed lines.

Figures 2(b-d) present the percentage depth dose (PDD) curves for 2×2 cm^2^ beamlets for different conditions studied [Figure 2a]. Figures 2b, c and d demonstrate the trends in dose reductions in the different interface regions of a 3×3×3 cm^3^ air-cavity for a single 2×2 cm^2^ field irradiation with Co-60, 6 MV and 15 MV beamlets, respectively. Condition A [Figure 2a] provides a geometry where a radiation beamlet passes through a homogenous water phantom; the dose calculations along the central axis (CAX) of the beamlet provide reference values for comparing the dose calculations in conditions B, C and D [Figure 2]. Condition B [Figure 2a], represents the geometry where CAX of the beamlet is situated in water at 0.025 cm distance from the air-water interface. The dose calculations along the CAX of the beamlet provide the dose reduction in water (or tissue) just beside the cavity. Thus, condition B essentially demonstrates the dose reductions in water predominantly due to the lateral electronic disequilibrium near the interface. In condition C, the CAX of the beamlet is situated at 0.025 cm from the air-water interface just inside the cavity. The condition C [Figure 2a] shows dose perturbations near the interface due to the combined effects of lateral electronic disequilibrium as well as re-establishment of buildup. Condition D [Figure 2a] represents the geometry where CAX of the beamlet passes through the centre of the cavity. Thus, dose calculations from the condition D show dose reductions in the proximal and distal interface regions, predominantly due to secondary buildup. A comparison of PDD curves in Figures 2(b-d) reveals significantly smaller dose reductions in lateral and distal interface regions for the Co-60 beamlets compared to the 6 MV and 15 MV beamlets.

### Overall interface percentage dose reduction (%DR) estimates

**Figure 3:**
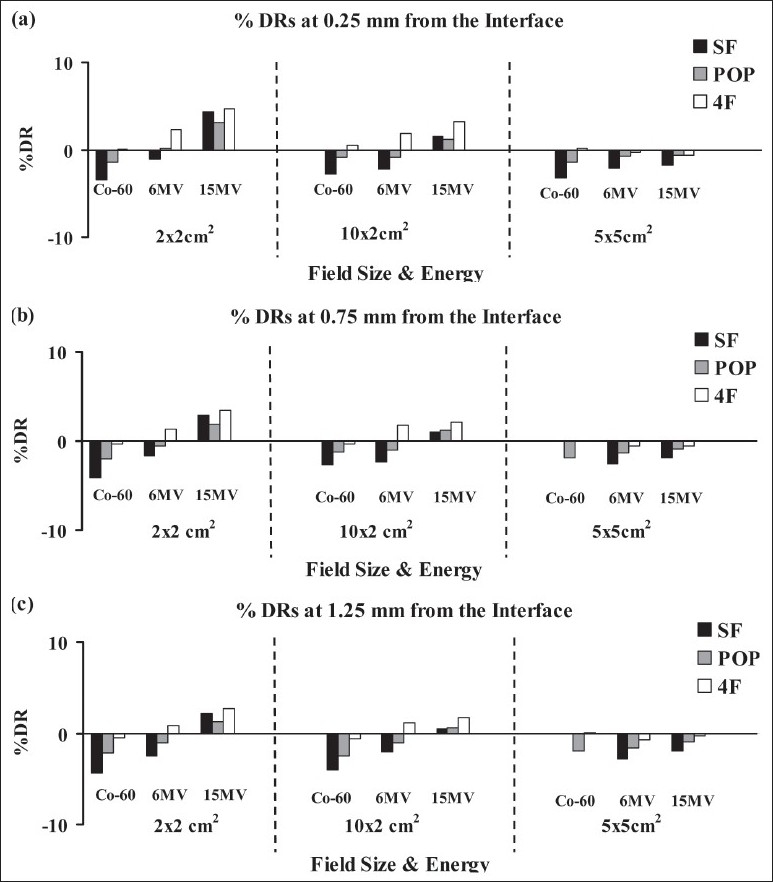
Comparison of percentage dose estimates (%DRs) in water, at (a) 0.25 mm (b) 0.75 mm and (c) 1.25 mm from the air-water interfaces for single field (SF), parallel opposed (POP) and four field (4F) techniques for 1×1×1 cm^3^ air cavity. Please see Figure 1 for the “dose points” indicated to represent the location of the interface dose calculation points (at 0.25, 0.75 and 1.25 mm from the interface (not to scale)).

**Figure 4:**
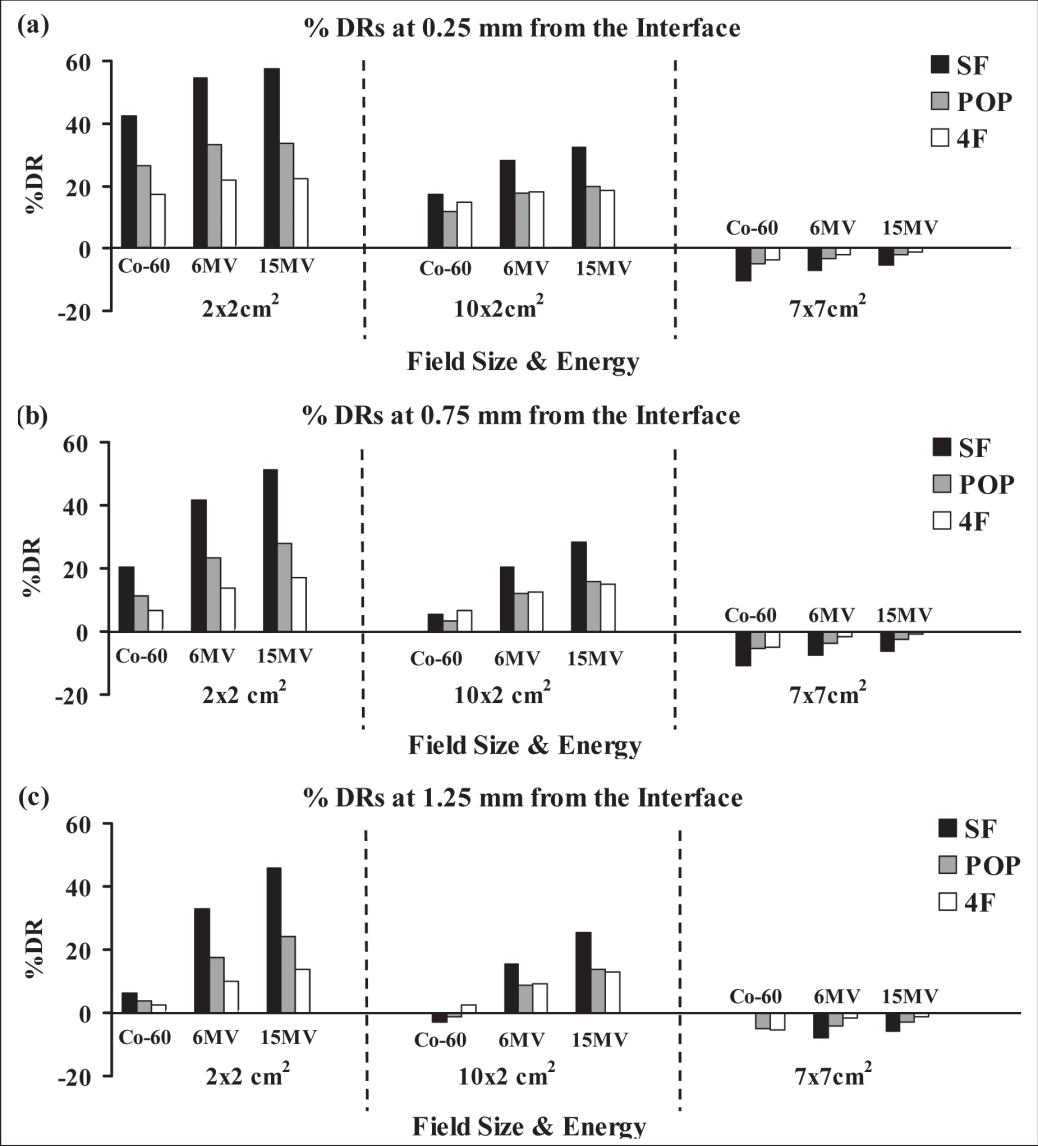
Comparison of percentage dose estimates (% DRs) in water, at (a) 0.25 mm (b) 0.75 mm and (c) 1.25 mm from the air-water interfaces for single field (SF), parallel opposed (POP) and four field (4F) techniques for 3×3×3 cm^3^ air cavity. Please see Figure 1 for the "dose points" indicated to represent the location of the interface dose calculation points (at 0.25, 0.75 and 1.25 mm from the interface (not to scale)).

For the 1×1×1 cm^3^ air-cavity the %DR estimates at 0.25 mm, 0.75 mm and 1.25 mm from the air-water interface are presented in Figures 3a, b and c, respectively, for different photon energies, field sizes and irradiation techniques. Similarly, the %DR estimates at different distances from the air-water interface for the 3×3×3 cm^3^ air-cavity are presented in Figure 4. The data presented in Figures 3 and 4 show that the %DR increases with the size of the air cavity, and the dose reductions are most severe for the smallest beam size (2×2 m^2^) at 15 MV. The data presented in Figure 4 show that dose reductions near the air-water interfaces are most severe for a single field and least severe for four-field irradiations. The dose reductions become more severe with increasing photon energy. In all, the dose reductions at larger distances from the interface, e.g. at 1.25 mm, are considerably higher for 6 and 15 MV beams than that for Co-60 beams [Figures 3 and 4]. This should be noted that negative values of % dose reductions (%DR) indicate % dose enhancements. These dose enhancements are due to higher gain in dose near interfaces from lack of attenuation because of the presence of air cavity compared to the dose reduction from electronic disequilibrium.

For the 1×1×1 cm^3^ cavity, no dose reductions in the interface region were seen for Co-60 for all beam sizes and techniques [Figure 3]. Irradiation of the same cavity with 6 MV showed < 2% dose reductions for only the 4 field technique, and no dose reductions were seen for SF and POP techniques. All beam sizes and techniques showed < 5%, < 4% and < 3% dose reductions at 0.25, 0.75 and 1.25 mm from the interface, respectively for 15 MV irradiations [Figure 3].

Typically, dose reductions at 0.25 mm from the interface for 2×2 cm^2^ beamlets of 6 MV photons were 54%, 33% and 22% for single field (SF), parallel opposed (POP) and four field (4F) irradiations, respectively [Figure 4a]. For the POP field irradiation with 10×2 cm^2^ fan beam, the %DRs at 0.25 mm from the distal interface were 11%, 18% and 20% for irradiations with Co-60, 6 MV and 15 MV photons, respectively [Figure 4a].

The Co-60 beamlets show significantly smaller dose reductions than the 6 MV and 15 MV beamlets at 1.25 mm from the interface than those at 0.25 mm and 0.75 mm from the interface [Figure 4]. Data presented in Figure 3c show %DRs at 1.25 mm from the interface for POP field irradiations with Co-60, 6 MV and 15 MV beams; which are 4%, 18% and 24% for 2×2 cm^2^; and, −1%, 9% and 14% for 10×2 cm^2^ fields, respectively.

The electronic disequilibrium near the air-water interface (on the water side of the interface) in the presence of an air cavity/gap takes place due to decrease in total photon and electron energy fluence compared to the total energy fluence in the same region in a homogeneous water phantom. This is consistent with the observation by Li *et al,*[2] that a decrease in fluence in the interface region is more severe for larger air cavities/gap. This also accounts for smaller %DR in the interface region of 1×1×1 cm^3^ air cavity than that of 3×3×3 cm^3^ air cavity.

The dose reductions were less severe or non-existent for the large 7×7 cm^2^ field size that encompasses the 3×3×3 cm^3^ air-cavity cavity with a 2 cm margin [Figure 4]. For example, the %DRs at 0.25 mm from the interface for POP irradiations with 15 MV photons were 34%, 20% and −2% for 2×2 cm^2^, 10×2 cm^2^ and 7×7 cm^2^ fields, respectively [Figure 4]. Similar patterns were also seen for the Co-60 and 6 MV beams. Our calculations show no dose reductions in the interface regions for the large field sizes encompassing the cavities with 2 cm margins (for 5×5 and 7×7 cm^2^ fields) for all techniques and beam energies [Figures 3 and 4].

## Discussion

In IMRT/tomotherapy treatment planning, the effect of dose reductions in the interface regions around air cavities could be potentially considered and compensated for during the optimization and inverse planning process. However, dose calculation algorithms of many commercial treatment planning systems (TPS) encounter severe limitations in the conditions of electronic disequilibrium,[23–25] particularly in IMRT techniques.[26–28] The issues related to the accuracy of dose calculations near air-cavities in the head and neck region has been discussed in many recent studies.[24,26–28] Majority of the commercial treatment planning systems recommend a minimum dimension of 0.25 cm for the dose calculation matrix in clinical IMRT/tomotherapy treatment planning. The voxel size of the dose calculation matrix, as well as its spatial relationship with the irregular shaped cavities, further limits the TPS's ability to accurately calculate the doses at sub-millimeter distances from the air-tissue interfaces. To help understand the issues related to dose near such interfaces, we adopted simple cubic cavity geometries in water phantom in this study using Monte Carlo simulations [Figure 1]. This simple geometry allowed us to accurately investigate the extent of dose reductions in air-water (or air-tissue) interface regions. For example, the choice of a simple cubic cavity geometry was instrumental in clearly demonstrating the extent of dose reduction entirely in the water near the interface (see curve B in Figures 2 b, c and d), as well as in the proximal and distal interface regions for a 2×2 cm^2^ beamlet [Figure 2]. Similar geometries enabled us to provide the detailed trends in dose reductions near interfaces for different beams, techniques and cavity sizes [Figures 3 and 4].

Our results show that the magnitude of dose reduction near air-water interfaces decreases with the increase in the number and size of radiation beams and increases with photon energy. In this work, we have used a maximum of four beam irradiations. However, in a realistic tomotherapy or IMRT dose delivery, a combination of multiple number of sub-fields within fields from many beam directions is employed. Thus, the magnitude of interface dose reductions will likely be smaller than those for the limited number of radiation fields used in this study. However, with the increasing use of techniques employing small field irradiations (tomotherapy and IMRT) the interface dose reductions may still remain relevant in the context of local control of lesions situated in the close vicinity of air cavities. The authors are currently in the process performing more rigorous investigations of interface doses near air-cavities for realistic tomotherapy and IMRT treatment plans using actual patient CT images. These investigations will involve MC dose calculations in finer volume elements surrounding air cavities. This will provide more accurate estimates of air-tissue interface dose reductions in IMRT/tomotherapy treatments.

## Conclusions

Significant dose reduction may occur near air-water (or air-tissue) interfaces for treatment techniques using small beams (beamlets) such as employed in IMRT and tomotherapy. The magnitude of dose reduction increases with the size of the air cavity and photon energy. Dose reductions in the interface region decrease with increase in the number of beams used in the treatment, particularly for irradiations with very small beams. No dose reductions were observed for situations where air cavities were irradiated with a field size that included 2 cm margins around cavity. This demonstrates the absence of interface dose reductions in conventional 3DCRT techniques. Regardless of field size, technique and photon energy, negligible or no interface dose reductions were seen for 1×1×1 cm^3^ air cavities. The volume of under-dosed water in the interface region is higher at higher photon energies. For the geometries investigated in this study, Co-60 beams showed significantly smaller dose reductions in the interface region than that obtained for 6 MV and 15 MV beams. This shows the potential advantage of Co-60 based tomotherapy and IMRT applications in situations where malignant disease is wrapped around or situated in the close vicinity of natural or artificial air-cavities such as in head and neck cancers.
